# Osteopontin enhances multi-walled carbon nanotube-triggered lung fibrosis by promoting TGF-β1 activation and myofibroblast differentiation

**DOI:** 10.1186/s12989-017-0198-0

**Published:** 2017-06-08

**Authors:** Jie Dong, Qiang Ma

**Affiliations:** Receptor Biology Laboratory, Toxicology and Molecular Biology Branch, Health Effects Laboratory Division, National Institute for Occupational Safety and Health, Centers for Disease Control and Prevention, Mailstop 3014, 1095 Willowdale Road, Morgantown, WV 26505 USA

**Keywords:** Osteopontin, Multi-walled carbon nanotube, Fibrosis, TGF-β1 signaling, Myofibroblast, Fibroblast

## Abstract

**Background:**

Carbon nanotubes (CNTs) have been used in a variety of applications because of their unique properties and functions. However, many CNTs have been shown to induce lung fibrosis in experimental animals with some at a potency greater than that of silica, raising concern over possible toxic effects of CNT exposure in humans. Research into the mechanisms by which CNTs induce pulmonary fibrosis is warranted in order to facilitate the understanding, monitoring, and treatment of CNT-induced lung lesions that might occur in exposed populations. The current study focuses on investigating the role of osteopontin (OPN) in the development of lung fibrosis upon exposure to multi-walled carbon nanotubes (MWCNTs).

**Methods:**

C57BL/6J (WT) and Opn knockout (KO) mice were exposed to MWCNTs by pharyngeal aspiration to examine the acute and chronic effects of MWCNT exposure. The role of OPN and its mode of action in lung fibrosis development were analyzed at the cellular and molecular levels in vivo and in vitro.

**Results:**

OPN was highly and persistently induced in both the acute and chronic phases of the response to MWCNT exposure in mouse lungs. Comparison between WT and Opn KO mice revealed that OPN critically regulated MWCNT-induced lung fibrosis as indicated by reduced fibrotic focus formation and myofibroblast accumulation in Opn KO lungs. At the molecular level, OPN promotes the expression and activation of TGF-β1, stimulates the differentiation of myofibroblasts from fibroblasts, and increases the production of fibrous matrix proteins in lungs and cultured lung cells exposed to MWCNTs.

**Conclusion:**

OPN is highly induced in CNT-exposed lungs and plays critical roles in TGF-β1 signaling activation and myofibroblast differentiation to promote fibrosis development from MWCNT exposure. This study reveals an OPN-dependent mechanism to promote MWCNT-induced lung fibrosis. The findings raise the possibility of using OPN as a biomarker to monitor CNT exposure and as a drug target to halt fibrosis development.

## Background

Carbon nanotubes (CNTs) have been developed as nanomaterials with unique properties and functions enabling them for a broad range of industrial and commercial applications. Production and utility of CNT-containing materials have been increased rapidly in recent decades [1]. However, some CNT materials are predicted to have adverse health impacts on exposed populations, because their physicochemical properties, such as nano-scaled size, fiber-like shape, high respirability, and apparent biopersistence, have been associated with fibrosis and cancer, and some pathologic effects of CNTs have been confirmed in laboratory animals [2–4].

Fibrosis commonly occurs in the lungs of animals exposed to CNTs of various types [5–7]. Notably, the fibrotic lesions bear a high degree of similarity to pneumoconiosis and to idiopathic pulmonary fibrosis (IPF), human lung fibrosing diseases that are frequently progressive and incurable. CNT-induced lung fibrosis appears to reflect the tissue response to foreign body deposition in the lungs. The lesion initiates as an acute response manifesting rapid-onset inflammatory infiltration, induction of cytokines, growth factors, and extracellular matrix (ECM) proteins, and formation of fibrotic foci, followed by partial resolution of the acute pathology but progression to chronic interstitial fibrosis and granuloma formation [8]. The mechanism(s) underlying CNT-induced lung fibrosis at the cellular and molecular levels remains largely elusive.

A hallmark of lung fibrosis is the excessive deposition of collagen fibers in the ECM and remodeling of the alveolar parenchyma to lead to progressive scarring and failure of the lungs. At the cellular level, fibrosis development is driven by the activation of fibroblasts and formation of myofibroblasts. Activated lung resident fibroblasts migrate to the site of foreign body deposition or tissue injury where they proliferate and differentiate into myofibroblasts. Myofibroblasts are characterized by their high capacity of ECM production and de novo synthesis of α-smooth muscle actin (α-SMA), which enable the cells to produce excessive amounts of collagens in fibrotic foci and to mediate the contraction of fibrosing tissues, respectively [8–10].

At the molecular level, the transforming growth factor-β1 (TGF-β1) has been identified as one of the most predominant endogenous regulators of fibrosis. Elevated expression of TGF-β1 is commonly detected in lung fibrotic lesions, such as IPF and bleomycin-induced lung fibrosis; and overexpression of active TGF-β1 leads to persisting lung fibrosis, whereas inhibition of TGF-β1 signaling by inhibiting or knocking out TGF-β receptors attenuates lung fibrosis in animal models [11–15]. TGF-β1 exhibits multiple pro-fibrotic activities; in particular, it markedly stimulates fibroblast proliferation and the fibroblast-to-myofibroblast differentiation to directly modulate fibrosis development [9, 10, 16–18]. TGF-β1 is secreted into the ECM in a latent complex form from activated macrophages and fibroblasts. Upon stimulation, latent TGF-β1 is activated to release functional TGF-β1, which binds to its receptors on the cell surface and induces the transcription of major fibrotic genes through the Smad-dependent pathway. TGF-β1 also activates Smad-independent pathways, such as the PI3K-AKT signaling, to regulate pro-fibrotic activities including myofibroblast formation [16, 18, 19].

Osteopontin (OPN; secreted phosphoprotein 1 or SPP1) is a glycoprotein secreted by a number of types of cells including inflammatory, immune, fibroblast, osteoblast, and cancer cells [20]. As both a cytokine and an ECM protein, OPN regulates a range of physiologic and pathologic processes, such as inflammatory infiltration, tissue remodeling, bone remodeling, and cancer metastasis [21, 22]. OPN exhibits a high level of expression in wound healing and is highly induced during organ fibrosis in animal models with significant pro-fibrotic activities [23–34]. OPN is also induced in the lungs upon prolonged exposure to MWCNTs or single-walled carbon nanotubes (SWCNTs) [35–40]. Moreover, OPN appears to be activated in the biofluids of MWCNT-exposed workers [41]. Whether OPN plays a role in CNT-induced lung pathology, in particular, lung fibrosis, remains to be examined.

Given the multiple roles of OPN in tissue fibrosis, we attempted to characterize OPN expression and analyze the functions of OPN, if any, in lung fibrosis induced by MWCNTs. We found that OPN is highly and persistently induced by MWCNTs in mouse lungs during both the acute and chronic phases of fibrosis development, implicating a role of OPN in both the initiation and the progression of lung fibrosis. By utilizing Opn knockout (KO) mice and cultured primary lung cells, we demonstrated that OPN promotes MWCNT-induced lung fibrosis through the activation of TGF-β1 signaling and promotion of myofibroblast differentiation and activation in the lungs. This study identifies an OPN-dependent mechanism that boosts MWCNT-induced lung fibrosis.

## Methods

### Multi-walled carbon nanotubes

MWCNTs were obtained from Mitsui & Company (XNRI MWNT-7, lot #05072001K28, Tokyo, Japan). A dispersion medium (DM), containing 0.6 mg/ml mouse serum albumin (Sigma-Aldrich, St. Louis, MO, USA) and 0.01 mg/ml 1,2-dipalmitoyl-sn-glycerol-3-phosphocholine (Sigma-Aldrich) in Ca^2+^- and Mg^2+^-free PBS, pH 7.4, was used to disperse MWCNTs and as the vehicle control [6, 42]. DM and MWCNT suspension were freshly prepared before use.

The characteristics of these MWCNTs have been reported previously [42]. The MWCNTs have a count mean diameter of 49 nm with a normal distribution and a standard deviation of 13.4 nm. The length distribution follows a log-normal distribution with a median length of 3.86 μm and a geometric standard deviation of 1.94 μm. Trace element contaminations are at low levels, i.e., 0.78% for all metals, 0.41% for sodium, and 0.32% for iron. The number of the walls ranges from 20 to 50 for each tube and the average surface area determined by nitrogen absorption-desorption technique is 26 m^2^/g. There is a dominant C 1s peak on X-ray photoelectron spectroscopy analysis, indicating sp^2^-hybridized graphite-like carbons as the majority of the structure. A minor component of C-O bond is detected, suggesting the formation of some hydroxyl groups on the surface of the MWCNTs.

The physicochemical properties of XNRI MWNT-7 have been associated with the biological activities of the MWCNTs to a certain extent. Compared with many CNTs that are long and slender, XNRI MWNT-7 CNTs are short and rod-like. This unique shape and rigidity enable the fibers to penetrate lung structures, such as alveolar walls, and migrate to distant locations, such as the pleural space [43]. Whether and how the rod-shape and rigidity of the MWCNT fibers contribute to their fibrogenicity remain debatable. It has been shown that the fibrogenic activity of CNT fibers correlates primarily with the number of individual fibers or small CNT clusters distributed in the interstitial space, which does not appear to depend on the rigidity and rod-like shape of the fibers, because non-rigid CNTs, such as SWCNTs, can distribute significantly more individual fibers in the alveolar interstitial space and consequently cause greater fibrogenic effects than XNRI MWNT-7 on an equal mass basis administered via the same route [3, 43]. In this regard, SWCNTs appear to be more potent to induce fibrosis than the MWCNTs independently of the rigidity of the fibers.

The fibrogenic activity of rod-like MWCNTs was compared with that of crocidolite asbestos, revealing that both inducers, when administered at similar doses, elicited inflammation and fibrotic lesions in mouse lungs with comparable magnitudes and time courses [44, 45]. Similar fibrogenic effects were also observed between SWCNTs and crocidolite asbestos at 1 year post-exposure [46]. Additionally, XNRI MWNT-7 CNTs appear to induce lung fibrosis at a greater potency than crystalline silica [6, 30]. Taken together, these findings imply that fibrogenic CNTs share considerable similarities with each other on their fibrogenicity, which is determined by their ability to distribute individual fibers in lung tissues to a large extent. This notion is also reflective of the fibrogenic effects of respirable asbestos fibers and silica particles. Nevertheless, differences in the biological effects and their underlying mechanisms among various CNTs and respirable particles and fibers caused by their unique physicochemical properties are well expected and warrant full consideration when comparing across fibrogenic inducers.

The relatively large specific surface area with some hydroxyl modifications of XNRI MWNT-7 suggests that there is a certain level of bonding strength between the fibers that is sometimes expressed as the bonding index or BIN. This bonding capacity would affect the physical state and properties of carbon nanotubes in solution and in tissues, including the agglomeration or aggregation of fibers, as well as the release of individual fibers from larger fiber clusters over time. Well-dispersed XNRI MWNT-7 CNTs administered by either pharyngeal aspiration or inhalation tend to have a large portion in the form of large MWCNT structures that contain more than four fibers in number and account for more than half of the initial lung burden [47]. These larger structures are often distributed in the lung macrophage space and can be cleared through alveolar macrophages rapidly. On the other hand, the amount of singlet fibers and smaller CNT structures present in the interstitial space appears to remain relatively constant, or even increase, over time. This finding suggests that, in addition to being cleared by alveolar macrophages, larger MWCNT structures undergo disassociation to release singlet and small clumps of fibers into the tissues, which in part reflects the inter-fiber bonding strength or BIN of the MWCNTs.

### Animals and treatment

Eight- to 10-week-old male C57BL/6J (WT) and Opn KO (B6.129S6(Cg)-*Spp1* ^*tm1Blh*^/J, Opn−/−) mice were purchased from The Jackson Laboratory (Bar Harbor, ME, USA). The mice were maintained in an accredited, specific pathogen-free and environmentally controlled facility at the National Institute for Occupational Safety and Health. All experiments involving animals were approved by the Institutional Animal Care and Use Committee.

A single dose of 40 μg MWCNTs in 50 μl of DM was administered by oropharyngeal aspiration, which is a noninvasive route of administration to deliver a specific dose of respirable materials, such as CNTs, into animal lungs to result in an even distribution of the materials in the lungs [42, 48]. This dose has been shown to induce both acute and chronic inflammatory and fibrotic responses to CNT exposure to a significant level in mouse lungs [6, 42]. The MWCNTs were used to compare between oropharyngeal aspiration and inhalation for pulmonary effects [47]. At nearly equivalent lung burdens of the MWCNTs, lung inflammation on day 1 post-exposure was similar between a single dose exposure by aspiration at a dose of 80 μg/mouse and by aerosol inhalation at 5 mg/m^3^, 5 h/day, 12 days. This inhalation paradigm results in a lung burden in the mouse equal to a predicted human lung burden on an equivalent alveolar surface area basis for a person performing light work at 7 μg/m^3^ for 13 years. XNRI MWNT-7 CNTs at 40 or 80 μg are known to produce comparable pulmonary effects [42]. Thus, a dose of XNRI MWNT-7 at 40 or 80 μg by aspiration in mice would produce an initial lung burden in humans comparable to that by inhalation.

The inflammatory response to the inhalation exposure became greater than that to aspiration exposure by ~4-fold, possibly caused by the faster clearance of CNT structures and resolution of inflammation in aspiration-exposed lungs than those in inhalation-exposed lungs. It was observed that a single dose exposure by aspiration would result in more large CNT structures in the airways and alveolar space than inhalation exposure. In part, this is due to the rapid delivery and accumulation of CNTs on the surface of airways and alveolar structures by aspiration, compared with the low level, continuous exposure over a period of time by inhalation. As discussed above, a portion of large CNT structures are cleared through alveolar macrophages rapidly. Large CNT structures also disassociate to release singlet or small clusters of CNT fibers over time to result in a relatively stable amount of individual fibers in the alveolar tissue to stimulate interstitial fibrosis development. These findings support the notion that XNRI MWNT-7 CNTs at 40 to 80 μg/mouse via pharyngeal aspiration produce sufficient lung burdens to induce fibrosis in mouse lungs, which is relevant to inhalation administration in mice and to possible human exposure, with respect to lung burden and lung fibrosis development.

### Primary mouse lung fibroblast culture and treatment

Fresh lung tissues from eight-week-old C57BL/6J mice were promptly excised after euthanasia, washed with PBS, and cut into 1 mm^3^-sized tissue pieces. The pieces were then suspended in Dulbecco’s Modified Eagle’s Medium (DMEM, Thermo Fisher Scientific, Waltham, MA, USA) containing 10% Fetal Bovine Serum (FBS, Thermo Fisher Scientific) and 1× Antibiotic-Antimycotic (Thermo Fisher Scientific), and cultured on plate at 37 °C in a humidified 5% CO_2_ incubator. The culture medium was changed every three days. When the fibroblasts reached 80% confluence on the plate, cells were passaged at 1:5 dilution. The fibroblasts from passages five to eight were used for experiments. The cells were seeded and cultured in serum-free DMEM for 24 h before being treated with one or a combination of the following agents: DM, 2 μg/ml MWCNTs, 5 μg/ml MWCNTs, OPN neutralizing antibodies (R&D Systems, Minneapolis, MN, USA, 1 μg/ml), TGF-β1 neutralizing antibodies (R&D Systems, 1 μg/ml), and TGF-β receptor inhibitor SB525334 (Selleck Chemicals, Houston, TX, USA, 10 μM), in serum-free DMEM, for 24 h. The cells were examined for viability to show that no significant toxicity, such as cell death and degeneration, took place under the exposure. The cells were then analyzed using immunofluorescence and immunoblotting assays.

The treatment doses for the in vitro experiments were 2 and 5 μg/ml or 0.44 and 1.11 μg/cm^2^ surface area for MWCNTs. The rationale for choosing these doses is two-fold. First, under these doses, no apparent cellular toxicity, such as cell degeneration and cell death, is found; yet alterations in cellular signaling and gene expression can be observed and quantified. Second, these doses are relevant to in vivo doses that produce lung inflammatory and fibrotic responses in mice, and thus, are potentially useful for extrapolation of the in vitro findings to in vivo effects. The MWCNTs have been shown to stimulate pulmonary responses in a dose rage of 5 to 80 μg/mouse by oropharyngeal aspiration with prominent fibrotic effects at doses of 40 and 80 μg/mouse [6, 42]. The alveolar surface area of an adult mouse has recently been measured to be 82.2 cm^2^ by using a newly developed, efficient stereological method [49]. Therefore, the in vivo dose range of 5 to 80 μg/mouse is equivalent to a surface dose range of 0.061 to 0.973 μg/cm^2^ of the mouse lung alveolar surface area; and the doses of 0.44 and 1.11 μg/cm^2^ used in the in vitro experiments are within or close to this in vivo mouse dose range.

### Histopathology, immunohistochemistry and immunofluorescence

The left lung lobe was fixed with 10% neutral buffered formalin and embedded in paraffin. Sections of 5 μm thickness were used to perform Masson’s Trichrome staining and immunohistochemistry staining. Masson’s Trichrome staining was carried out following standard protocol for histopathological analysis, in which six samples per group were observed and evaluated. Fibrotic changes were quantified using the modified Ashcroft score with grades from 0 to 8 to represent from normal lung alveoli without fibrotic burden to complete obliteration of the alveolar space in a fibrotic mass within the microscopic field [50]. Immunohistochemistry was performed after deparaffinization, rehydration, and antigen retrieval, as described previously [30]. The primary antibodies used were anti-OPN (R&D Systems), anti-TGF-β1 (Santa Cruz, Dallas, TX, USA), and anti-p-Smad2/3 (Ser 423/425) (Santa Cruz). Images were photographed using Olympus Provis AX-70 system (Olympus, Center Valley, PA, USA). Positive staining was measured using the ImageJ program (National Institutes of Health, Bethesda, MD, USA) and the relative intensity was presented as the mean ± standard deviation (SD) with *n* = 4.

Cryostat sections from frozen left lung lobe (7 μm) or primary mouse lung fibroblasts cultured on four-well chamber slides were fixed with 4% paraformaldehyde and used for immunofluorescence as described previously [30]. Briefly, the slides were blocked for 1 h at room temperature, immunostained with primary antibodies at 4 °C overnight, incubated with Alexa Fluor 488- or Alexa Fluor 594-conjugated secondary antibodies (Thermo Fisher Scientific) for 1 h at room temperature in dark, and mounted with ProLong Diamond Antifade Mountant with DAPI (Thermo Fisher Scientific). When mouse primary antibodies were applied, the blocking reagent and antibody diluent from the M.O.M. Immunodetection Kit (Vector Laboratories, Burlingame, CA, USA) were used to eliminate background staining. The primary antibodies used for immunofluorescence were anti-Collagen I (Abcam, Cambridge, MA, USA), anti-FN1 (Abcam), anti-Hsp47 (EMD Millipore, Billerica, MA, USA), anti-Vimentin (Santa Cruz), anti-α-SMA (Abcam or Sigma-Aldrich), anti-PDGFR-β (Abcam), anti-Phospho-Smad2 (Ser465/467)/Smad3 (Ser423/425) (Cell Signaling Technology, Danvers, MA, USA), anti-OPN (R&D Systems), and anti-TGF-β1 (Santa Cruz) antibodies. Images were taken with a Zeiss LSM 780 confocal microscope (Carl Zeiss Microscopy, Jena, Germany). Quantification of positive staining was performed using the ImageJ program to derive the relative intensity shown as the mean ± SD (*n* = 4).

### Quantitative RT-PCR (qRT-PCR)

Total RNA was extracted from lung tissues using RNeasy Mini Kit (QIAGEN, Valencia, CA, USA). qRT-PCR was performed with RT^2^ SYBR Green ROX qPCR Mastermix (QIAGEN), as described previously [6]. Mouse glyceraldehyde 3-phosphate dehydrogenase (*Gapdh*) was used as an internal control for normalization. Primer sequences are available upon request. Fold changes were presented as the mean ± SD (*n* = 4).

### Enzyme-linked immunosorbent assay (ELISA)

Bronchoalveolar lavage (BAL) fluid was obtained following the method described previously [6, 42]. Protein levels of OPN were determined using Mouse Osteopontin DuoSet ELISA kit (R&D Systems). Samples from five animals per group were measured to derive the mean ± SD.

### Immunoblotting

Randomly selected lung tissue samples were homogenized and lysed in T-PER Tissue Protein Extraction Reagent (Thermo Fisher Scientific), whereas cultured primary mouse lung fibroblasts were lysed in RIPA Lysis Buffer (Santa Cruz). Whole protein extract (10, 20, or 30 μg) was resolved on a 4–15% or 8–16% Criterion TGX Gel (Bio-Rad, Hercules, CA, USA). Actin was examined as a loading control. Representative blotting images were presented. The primary antibodies used for immunoblotting were anti-OPN (R&D Systems), anti-FN1 (Abcam), anti-FSP1 (EMD Millipore), anti-TGF-β1 (Santa Cruz), anti-Phospho-Smad2 (Ser465/467)/Smad3 (Ser423/425) (Cell Signaling Technology), anti-Smad2/3 (Cell Signaling Technology), and anti-Actin (Santa Cruz) antibodies.

### Statistical analysis

The statistical analysis of differences between experimental groups was performed with one-way ANOVA followed by between group comparisons using standard procedures. Major quantitative experiments were repeated at least once, and representative data were presented as the mean ± SD. A *p* value of less than 0.05 was considered statistically significant. *, *p* < 0.05; **, *p* < 0.01; and ***, *p* < 0.001.

## Results

### MWCNTs induce rapid and prolonged expression and secretion of OPN in mouse lungs

**Fig. 1:**
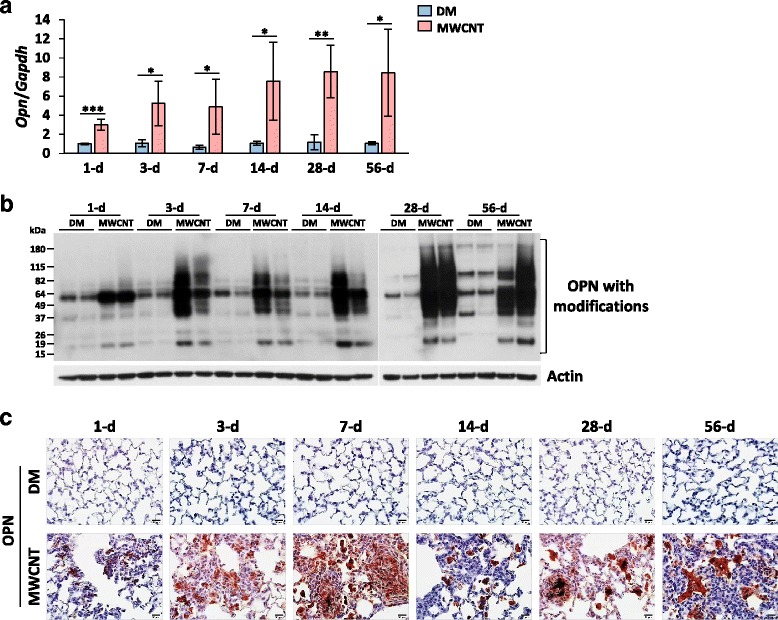
Induction of OPN by MWCNTs. WT mice received DM or MWCNTs (40 μg) and were sacrificed on days 1, 3, 7, 14, 28, and 56 post-exposure. (**a**) qRT-PCR analysis of Opn mRNA in total RNA extracts from lung tissues (*n* = 4). *Gapdh* gene was used as the internal control. (**b**) Immunoblotting of OPN in lung tissue whole protein lysates (*n* = 2). Lung proteins from randomly selected samples of each group were studied, and a representative blotting image is presented. OPN protein with post-translational modifications was detected. Actin protein was used as the loading control. (**c**) Immunohistochemistry of OPN on lung tissue sections. Red indicates positive staining of OPN, and blue indicates nuclear counterstaining (scale bar: 20 μm)

To analyze the role of OPN in the pulmonary effects of CNT exposure, we first examined the expression pattern of OPN in mouse lungs exposed to MWCNTs. Adult wild-type C57BL/6J mice were exposed to MWCNTs (40 μg) or DM (vehicle control) by pharyngeal aspiration. Lung tissues were examined on days 1, 3, 7, 14, 28, and 56 after treatment to recapitulate the time course of the early and the chronic phase responses. At all the time points examined, the expression of Opn mRNA was low in DM-treated lungs, but was significantly elevated in MWCNT-exposed lungs, starting with a 3.0-fold induction on day 1 and increasing to 5.2, 4.9, 7.6, 8.6, and 8.4-fold induction on days 3, 7, 14, 28, and 56, respectively (Fig. 1a). Therefore, MWCNTs induced the expression of Opn mRNA rapidly and persistently at high levels throughout the early and the chronic phases of responses. The OPN protein is known to undergo multiple post-translational modifications upon secretion and activation. Immunoblotting revealed that OPN was at a low level in DM-treated lungs, but the level was dramatically increased by MWCNTs (Fig. 1b). Induction of OPN protein was >3-fold on day 1 and became increasingly higher on days 3, 7, 14, 28, and 56. Notably, multiple OPN protein bands with altered mobility were detected in MWCNT-exposed lungs from day 3 and thereafter, compared with DM control. These changes in OPN mobility are likely caused by post-translational modifications of OPN protein, including phosphorylation, N-glycosylation, O-glycosylation, and proteolytic cleavage, which often mark the activation of OPN (Fig. 1b).

Expression and distribution of OPN protein in lung tissues were visualized by immunohistochemistry (Fig. 1c). OPN protein was barely detectable in DM-exposed lungs, but its level was increased on day 1, became dramatically higher on day 3, and reached a peak on day 7 post-exposure to MWCNTs. Induction of OPN protein was reduced on day 14, but was increased again on day 28 and persisted to day 56. The distribution of OPN in the lungs was distinct during different stages of fibrosis development. Induced OPN was observed mainly in the interstitial and alveolar macrophages on day 1 post-exposure, and in macrophages and interstitial fibrotic foci where MWCNTs deposited on days 3 and 7. On day 14, on which the acute inflammation is largely resolved, induced OPN was detected mainly in and around macrophages, which predominate the lesioned area. In the chronic phase (days 28 and 56), OPN protein was localized within the interstitial fibrotic foci, the granulomas, and the interstitial macrophages. This pattern of time-dependent expression and distribution of OPN induced by MWCNTs correlates with the biphasic development of fibrosis and the locality of MWCNT-induced lesions in the lungs, suggesting that OPN plays certain roles in both the early and the chronic phases of lung fibrosis development induced by MWCNTs.

**Fig. 2:**
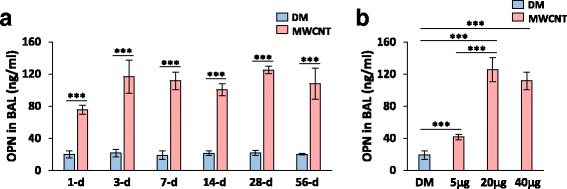
Induced secretion of OPN in the BAL. (**a**) Time course study. WT mice were treated with DM or MWCNTs (40 μg) for 1, 3, 7, 14, 28, and 56 days. The OPN level in BAL fluid was determined by ELISA (mean ± SD, *n* = 5). (**b**) Dose dependence study. WT mice were treated with DM or MWCNTs (5, 20, or 40 μg) for 7 days. The OPN level in BAL fluid was determined by ELISA (mean ± SD, *n* = 5)

The BAL fluid contains secreted proteins from stimulated lung cells that often reflect the underlying pathologic changes in the lungs. These proteins may also have functions at distant regions. OPN was detected by ELISA at a low level in the BAL fluid from DM-treated mice, but its level was significantly elevated in the BAL fluid from MWCNT-exposed mice (Fig. 2a). The level was increased by 3.8-fold upon treatment with MWCNTs on day 1 and was further boosted to 5.8-fold on day 3. High levels lasted through day 56 post-exposure, with a slight reduction on day 14 compared with those on days 3, 7, and 28. This trend of OPN secretion into the BAL qualitatively correlates with the expression pattern of OPN in lung tissues shown in Fig. 1c. Increased secretion of OPN into the BAL was dose-dependent. MWCNTs at a low dose (5 μg) induced a mild but significant elevation (2.2-fold) of the OPN level in the BAL on day 7 post-exposure, whereas treatment with 20 or 40 μg of MWCNTs for 7 days led to a striking increase in the amount of OPN in the BAL (6.6 and 5.9-fold, respectively) (Fig. 2b). Therefore, MWCNTs potently induced the secretion of OPN protein into the BAL.

Together, these data demonstrate that OPN expression in the lungs was rapidly and remarkably induced at both the mRNA and protein levels with concomitant marked secretion into the BAL fluid during both the early and the chronic phase responses to MWCNTs, which implicates both local and distant effects of OPN during the onset and the chronic progression of MWCNT-induced fibrosis development in the lungs.

### OPN promotes MWCNT-induced fibrotic changes in mouse lungs

**Fig. 3:**
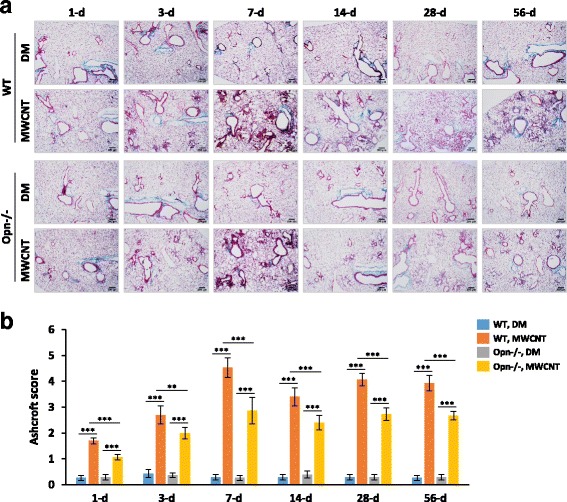
Attenuated fibrotic phenotypes in Opn KO lungs. WT and Opn KO mice were exposed to DM or MWCNTs (40 μg) for 1, 3, 7, 14, 28, and 56 days. (**a**) Histopathology and total collagen fibers were detected by Masson’s Trichrome staining of lung tissue sections, with collagen fibers stained blue (scale bar: 200 μm). Around a half of the left lung lobe is presented in each image to show and compare the overall changes in histopathology. Representative images for the treatment groups are presented. (**b**) Severity of pulmonary fibrosis was evaluated by modified Ashcroft score presented as mean ± SD (*n* = 6)

To examine the function of OPN during lung fibrosis development, the fibrotic phenotypes of WT and Opn KO (Opn−/−) lungs exposed to MWCNTs were compared. Fibrotic lesions were induced in WT lungs with a biphasic development compared with DM control. The early phase fibrotic and inflammatory lesions reached a peak on day 7, followed by resolution of the acute response on day 14 and progression to chronic fibrosis that was fully developed on day 28 and lasted to day 56 post-exposure. In the Opn KO lungs, exposure to MWCNTs also induced acute lesions that reached a peak on day 7; however, the lesions were at a much lower extent compared with WT, as assessed by the number and size of fibrotic foci (Fig. 3a). The acute response was resolved on day 14, but the conversion to chronic lesions was rather insignificant in Opn KO lungs compared with WT. It was noted that the DM-treated lungs from Opn KO mice also exhibited reduced staining compared with WT, suggesting that the lack of OPN alters the basal level morphology of the lungs to a certain extent. The fibrotic changes were quantified by using the Ashcroft score (Fig. 3b), which confirmed a statistically significant reduction of the fibrotic lesions in Opn KO lungs compared with WT at all the time points examined. Based on this time course of fibrosis development induced by MWCNTs, day 7 and day 28 were chosen as the time points to reflect the acute and the chronic phase responses, respectively, for subsequent analyses.

**Fig. 4:**
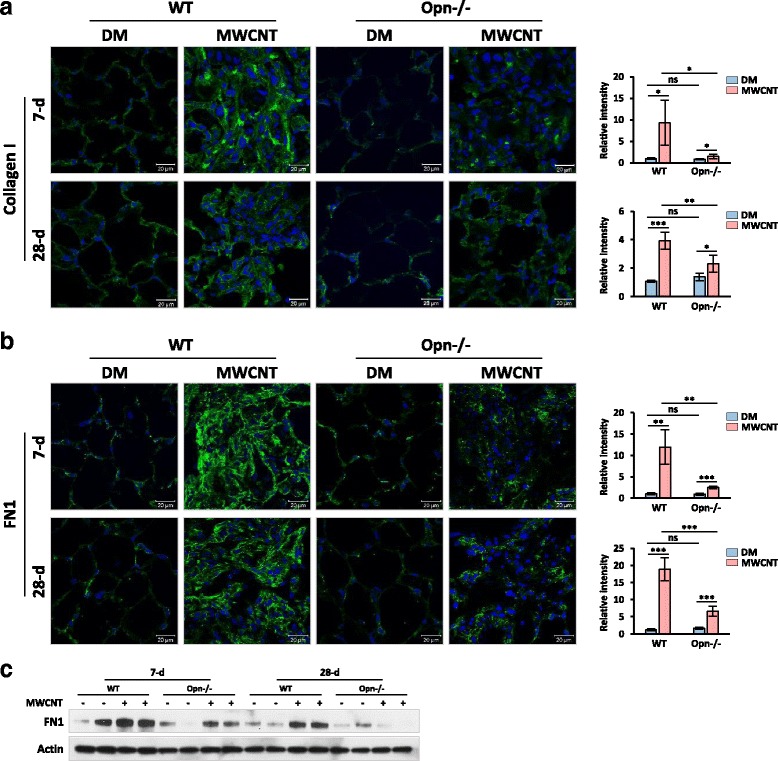
Quantification of fibrotic matrix protein expression. WT and Opn KO mice were exposed to DM or 40 μg MWCNTs for 7 or 28 days. (**a**) Immunofluorescence of Collagen I on mouse lung sections. (**b**) Immunofluorescence of FN1 on mouse lung sections. In (**a**) and (**b**), green indicates positive staining, and blue indicates nuclear staining (scale bar: 20 μm). Relative intensity of positive staining was measured and presented as mean ± SD (*n* = 4). (**c**) Immunoblotting of FN1 (*n* = 2). Actin was used as the loading control

The fibrotic lesions were also quantified by analyzing the expression and distribution of Collagen I and FN1, two major ECM proteins involved in the formation and remodeling of the fibrotic ECM, in the lungs. Both Collagen I and FN1 were detected at low levels in the interstitial space of DM-treated lungs from WT and Opn KO mice (Fig. 4a and b). MWCNTs drastically increased the expression and the interstitial deposition of Collagen I and FN1 in WT lungs on both day 7 and day 28 post-exposure, most prominently within fibrotic foci. The fold induction for Collagen I on day 7 and day 28 in WT lungs was 9.5 and 3.6, respectively (Fig. 4a), whereas that for FN1 was 11.2 and 15.7, respectively, over DM control (Fig. 4b). Induction of these two proteins was also observed in Opn KO lungs, but at significantly lower levels: the fold induction for Collagen I on day 7 and day 28 was 1.9 and 1.7, respectively (Fig. 4a), and that for FN1 was 2.8 and 4.1, respectively (Fig. 4b). We further examined the protein expression of FN1 in lung tissues by immunoblotting (Fig. 4c). The result revealed marked induction of FN1 protein on day 7 and day 28 in WT lungs consistently; but FN1 induction was substantially reduced on day 7 and nearly disappeared on day 28 in Opn KO lungs. These findings imply that OPN plays an important role in the ECM production and hence the fibrosis development in the lungs exposed to MWCNTs.

### OPN boosts fibroblast activation and myofibroblast differentiation elicited by MWCNTs in vivo

**Fig. 5:**
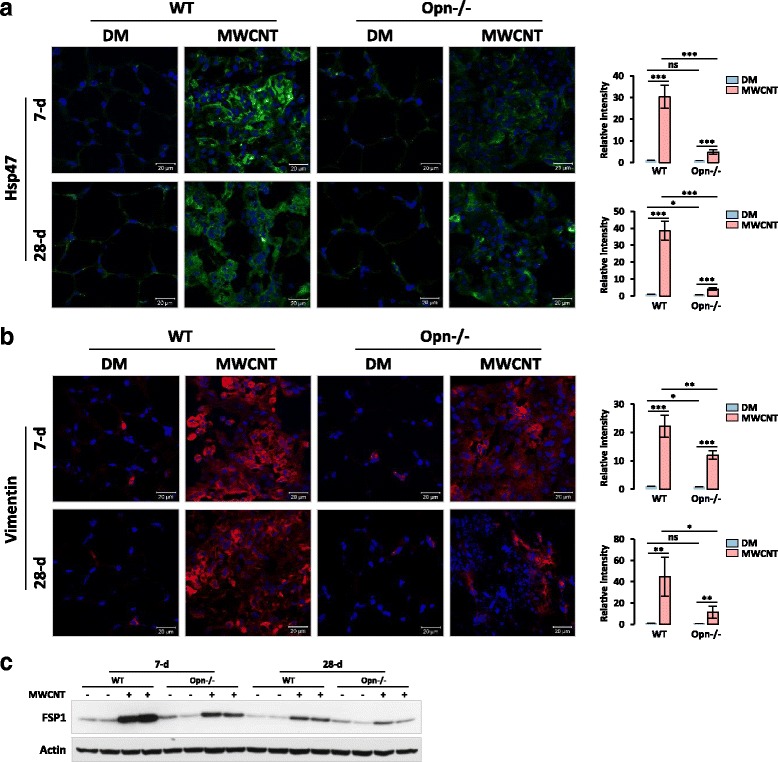
Fibroblast accumulation. WT and Opn KO mice were exposed to DM or 40 μg MWCNTs for 7 or 28 days. (**a**) Immunofluorescence of fibroblast marker Hsp47 on mouse lung sections. Green indicates positive staining, and blue indicates nuclear staining (scale bar: 20 μm). (**b**) Immunofluorescence of fibroblast marker Vimentin on mouse lung sections. Red indicates positive staining, and blue indicates nuclear staining (scale bar: 20 μm). In (**a**) and (**b**), relative intensity of positive staining was measured and presented as mean ± SD (*n* = 4). (**c**) Immunoblotting of fibroblast marker FSP1 (*n* = 2). Actin was used as the loading control

Formation of fibrotic foci is a prominent phenotype in MWCNT-induced lung fibrosis where fibroblasts are enriched and activated to differentiate into myofibroblasts. Myofibroblasts secrete a major portion of the fibrotic matrix proteins during fibrosis as well as mediate the contraction of the fibrosing tissue. Therefore, we examined the effect of Opn KO on fibroblasts and myofibroblasts in the lungs exposed to MWCNTs. We first visualized and quantified the accumulation of fibroblasts in fibrotic foci by immunofluorescence detection with antibodies specific for two fibroblast markers, Hsp47 (heat shock protein 47) and Vimentin. Positive staining for both Hsp47 (Fig. 5a) and Vimentin (Fig. 5b) was markedly increased by MWCNTs in the fibrotic foci of both WT and Opn KO lungs. However, the level of increase was apparently lower in Opn KO than WT lungs on both day 7 and day 28 post-exposure. Quantification of the relative fluorescence intensity of positive staining confirmed that the differences for induction between DM- and MWCNT-treated lungs and between MWCNT-treated WT and Opn KO lungs were statistically significant (Fig. 5a and b). The fold induction for Hsp47 on days 7 and 28 in WT was 29.2 and 37.5, respectively, but in Opn KO lungs, it was 4.8 and 5.6. The fold induction for Vimentin on days 7 and 28 in WT was 22.0 and 45.1, respectively, whereas in Opn KO lungs, it was 14.7 and 12.7. In a similar manner, the level of FSP1 (fibroblast specific protein 1), another fibroblast marker protein, was induced by MWCNTs in both WT and Opn KO lung tissues, but the induction was much lower in Opn KO than WT lungs, on both day 7 and day 28 post-exposure, as demonstrated by immunoblotting (Fig. 5c). Therefore, loss of OPN significantly reduced the accumulation and activation of fibroblasts in the lungs exposed to MWCNTs.

**Fig. 6:**
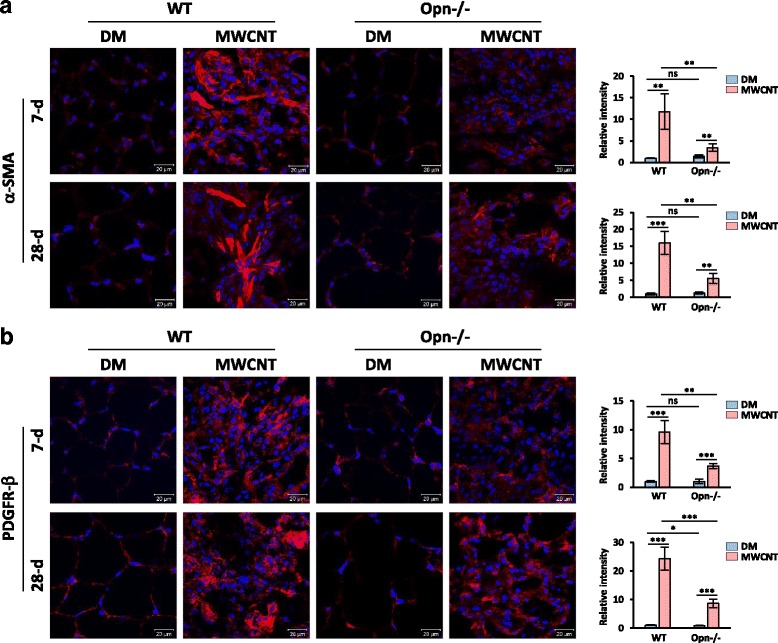
Myofibroblast differentiation. WT and Opn KO mice were exposed to DM or 40 μg MWCNTs for 7 or 28 days. (**a**) Immunofluorescence of myofibroblast marker α-SMA on mouse lung sections. (**b**) Immunofluorescence of myofibroblast marker PDGFR-β on mouse lung sections. In (**a**) and (**b**), red indicates positive staining, and blue indicates nuclear staining (scale bar: 20 μm). Relative intensity of positive staining was measured and presented as mean ± SD (*n* = 4)

α-SMA and the platelet-derived growth factor receptor-β (PDGFR-β) were used as markers for myofibroblasts to quantify myofibroblast formation. Positive staining for both α-SMA (Fig. 6a) and PDGFR-β (Fig. 6b) was dramatically increased by MWCNTs in the fibrotic foci of WT lungs, but was only slightly induced in the fibrotic foci of Opn KO lungs, on both day 7 and day 28 post-exposure. Quantification of the relative fluorescence intensity revealed that the fold induction for α-SMA on days 7 and 28 in WT was 11.6 and 16.9, respectively, but in Opn KO lungs, it was merely 2.4 and 4.4 (Fig. 6a). The fold induction for PDGFR-β on days 7 and 28 in WT was 9.5 and 24.3, respectively, whereas in Opn KO lungs, it was 3.6 and 10.5 (Fig. 6b). The difference between MWCNT-treated WT and Opn KO lungs was statistically significant for both α-SMA and PDGFR-β. Therefore, loss of OPN significantly reduced the number of myofibroblasts in the fibrotic foci, implicating OPN in myofibroblast differentiation in the lungs exposed to MWCNTs.

### OPN enhances the activation of TGF-β1 signaling by MWCNTs in mouse lungs

**Fig. 7:**
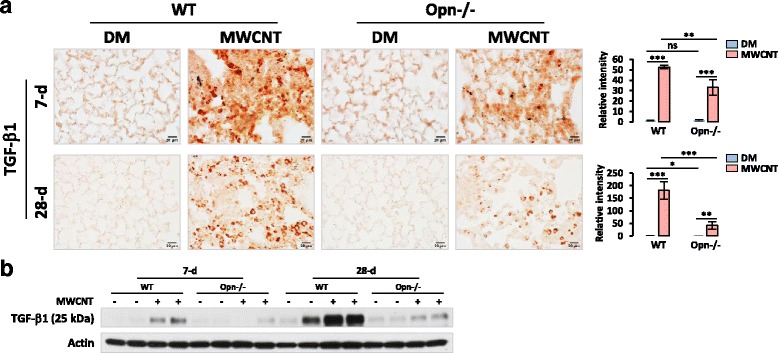
TGF-β1 expression and activation in the lungs. WT and Opn KO mice were exposed to DM or 40 μg MWCNTs for 7 or 28 days. (**a**) Immunohistochemistry of TGF-β1 on lung tissue sections. Red indicates positive staining of TGF-β1 (scale bar: 20 μm). Relative intensity of positive staining was measured and presented as mean ± SD (*n* = 4). (**b**) Immunoblotting of TGF-β1 (*n* = 2). Actin was used as the loading control

OPN cross-interacts with TGF-β1 signaling in several physiological and pathological processes, such as the epithelial to mesenchymal transition (EMT) during wound healing and the transformation of cancer stem cells to cancer-associated fibroblasts during tumorigenesis [51, 52]. Therefore, we tested if OPN modulates TGF-β1 activation and signaling to promote MWCNT-induced lung fibrosis. TGF-β1 protein was significantly induced in both WT and Opn KO lungs exposed to MWCNTs for 7 or 28 days, but the level of induction was significantly lower in Opn KO than WT lungs (Fig. 7a). Quantification of the relative intensity for TGF-β1 staining on days 7 and 28 confirmed this finding (Fig. 7a). Moreover, immunoblotting showed that the level of active TGF-β1, detected as the TGF-β1 protein band with an apparent molecular weight of ~25 kDa, was evidently elevated by MWCNTs in WT lungs on days 7 and 28 post-exposure, but this effect was not detected or was only mild in Opn KO lungs (Fig. 7b). These results demonstrate that OPN positively regulates the induction and activation of TGF-β1 by MWCNTs in the lungs during fibrosis development.

**Fig. 8:**
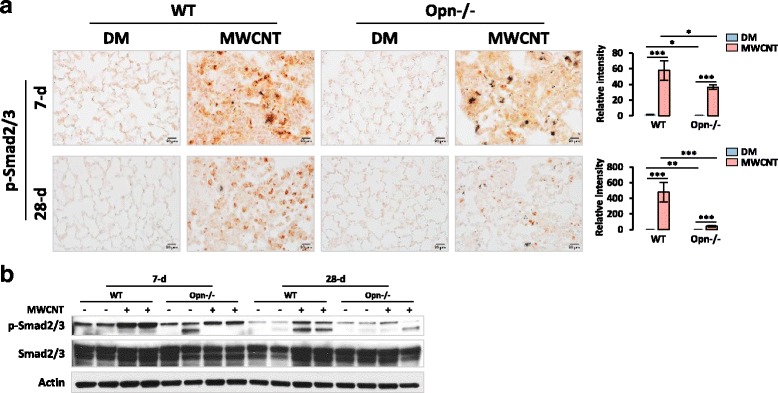
Activation of TGF-β1 signaling in the lungs. WT and Opn KO mice were exposed to DM or 40 μg MWCNTs for 7 or 28 days. (**a**) Immunohistochemistry of p-Smad2/3 on lung tissue sections. Red indicates positive staining of p-Smad2/3 (scale bar: 20 μm). Relative intensity of positive staining was measured and presented as mean ± SD (*n* = 4). (**b**) Immunoblotting of p-Smad2/3 and Smad2/3 (*n* = 2). On the blot for p-Smad2/3, the upper band is p-Smad2 and the lower band is p-Smad3; and on the blot for Smad2/3, the upper band is Smad2 and the lower band is Smad3. Actin was used as the loading control

Next, phosphorylation of Smad2/3 (p-Smad2/3), which indicates the activation of TGF-β1 signaling, was examined. Immunohistochemistry analysis on lung tissue sections clearly demonstrated that phosphorylation of Smad2/3 was significantly induced and the number of p-Smad2/3 positive cells was increased by MWCNTs in both WT and Opn KO lungs on days 7 and 28 post-exposure; but both increases were markedly reduced in Opn KO compared with WT lungs (Fig. 8a). Quantification of the p-Smad2/3 staining confirmed the significant differential induction of Smad2/3 phosphorylation in WT and Opn KO lungs by MWCNTs (Fig. 8a). We further analyzed the effect of Opn KO on the p-Smad2/3 level against total Smad2/3 protein by immunoblotting using whole lung protein lysates (Fig. 8b). The immunoblot analysis verified that MWCNTs induced the activation of TGF-β1 signaling in the lungs and OPN was required for the optimal induction, indicating OPN positively regulates both the expression and the activation of TGF-β1 in mouse lungs exposed to MWCNTs.

### OPN is required for activation of TGF-β1 signaling in fibroblasts and myofibroblasts in MWCNT-exposed lungs

**Fig. 9:**
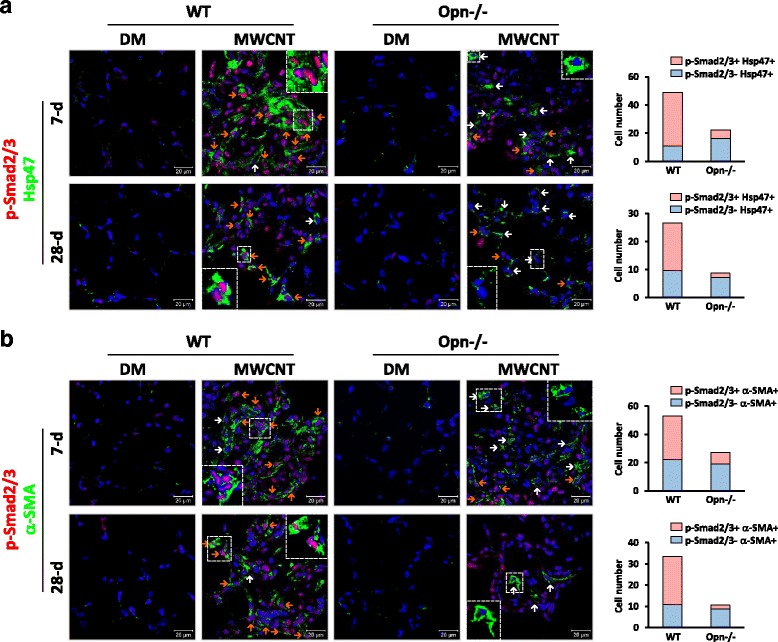
Activation of TGF-β1 signaling in lung fibroblasts and myofibroblasts in vivo. WT and Opn KO mice were exposed to DM or 40 μg MWCNTs for 7 or 28 days. (**a**) Induction and nuclear localization of p-Smad2/3 in fibroblasts were examined by double immunofluorescence staining of p-Smad2/3 (red) and Hsp47 (green). (**b**) Induction and nuclear localization of p-Smad2/3 in myofibroblasts were examined by double immunofluorescence staining of p-Smad2/3 (red) and α-SMA (green). In (**a**) and (**b**), blue indicates nuclear staining (scale bar: 20 μm). Representative fibroblasts or myofibroblasts that are positive for nuclear p-Smad2/3 (shown in purple color on the images from the overlapping of red and blue signals) are marked by orange arrows, and negative for nuclear p-Smad2/3 are marked by white arrows. The numbers of p-Smad2/3+ and p-Smad2/3- fibroblasts (Hsp47+) or myofibroblasts (α-SMA+) in MWCNT-exposed WT or Opn KO lungs were counted (*n* = 4) and presented beside the images

Because OPN critically modulates both the TGF-β1 signaling and the function of fibroblastic cells in MWCNT-induced lung fibrosis, it was posited that OPN stimulates the activation of TGF-β1 signaling directly in lung fibroblasts and myofibroblasts to promote fibrosis development. This notion was tested using double immunofluorescence staining of lung tissues. As shown in Fig. 9, the level of p-Smad2/3 was remarkably increased in WT lungs, as well as in Opn KO lungs albeit to a significantly reduced extent when compared with WT, on days 7 and 28 post-exposure to MWCNTs; this result is in agreement with the immunohistochemistry finding shown above (Fig. 8a). Moreover, a major portion of the p-Smad2/3 protein was found to be located in the nucleus, confirming the activation of the Smad-dependent TGF-β1 signaling pathway by MWCNTs in the lungs (see insets in Fig. 9a and b). Importantly, the induction and nuclear translocation of p-Smad2/3 were detected in the majority of Hsp47+ fibroblasts in MWCNT-exposed WT lungs on days 7 and 28 post-exposure (78% and 63%, respectively) (Fig. 9a). On the contrary, only a small portion of Hsp47+ fibroblasts in MWCNT-exposed Opn KO lungs showed nuclear staining of p-Smad2/3 (27% and 17%, respectively). Similarly, a high percentage of α-SMA+ myofibroblasts were positive for nuclear p-Smad2/3 staining in WT lungs on days 7 and 28 post-exposure to MWCNTs (58% and 68%, respectively); but in Opn KO lungs, only a small portion of α-SMA+ myofibroblasts showed positive staining of nuclear p-Smad2/3 (30% and 17%, respectively) (Fig. 9b).

These data clearly demonstrate that MWCNTs activate TGF-β1 signaling in fibroblasts and myofibroblasts of WT lungs, which may account for MWCNT-induced fibroblast activation, fibroblast-to-myofibroblast transformation, and myofibroblast activation. Loss of OPN impairs TGF-β1 signaling in both fibroblasts and myofibroblasts of MWCNT-exposed lungs, indicating that OPN is required for the activation of TGF-β1 signaling and function in lung fibroblastic cells by MWCNTs.

### OPN promotes MWCNT-induced fibroblastic response through TGF-β1 signaling in vitro

**Fig. 10:**
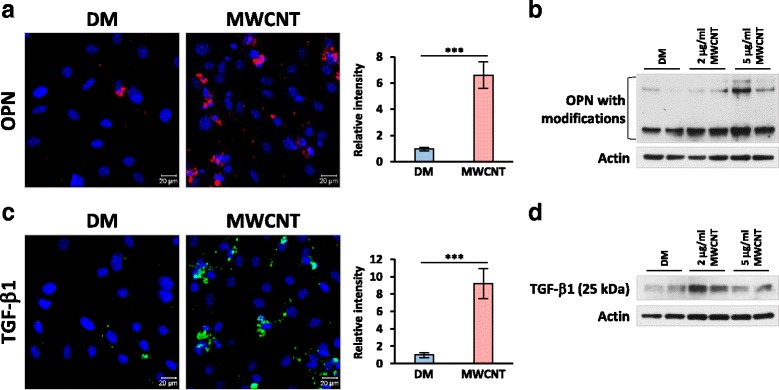
Induction of OPN and TGF-β1 expression and activation by MWCNTs in cultured lung fibroblasts. In (**a**) and (**c**), cells were treated with DM or 5 μg/ml MWCNTs for 24 h. OPN expression (**a**, red) and TGF-β1 expression (**c**, green) were determined by immunofluorescence. Blue indicates nuclear staining (scale bar: 20 μm). In (**b**) and (**d**), cells were treated with DM, 2 μg/ml MWCNTs, or 5 μg/ml MWCNTs for 24 h. Levels of OPN with post-translational modifications (**b**) and active TGF-β1 (**d**) were detected by immunoblotting (*n* = 2). Actin was used as the loading control

Regulation of TGF-β1 signaling in lung fibroblastic cells by OPN in vivo can result directly from its action on lung fibroblastic cells, or indirectly through other means, such as inflammatory cells that are activated by OPN and in turn modulate TGF-β1 signaling in fibroblasts and myofibroblasts. Therefore, a series of in vitro studies using primary fibroblasts derived from mouse lungs were performed to testify if OPN directly modulates TGF-β1 signaling in cultured fibroblasts. MWCNTs at a concentration of 5 μg/ml significantly induced the expression and secretion of OPN from cultured primary fibroblasts as shown by immunofluorescence staining (Fig. 10a). Induction of OPN by MWCNTs at concentrations of 2 and 5 μg/ml was further confirmed by immunoblotting, showing induction was dose-dependent (Fig. 10b). Similarly, immunofluorescence study showed that MWCNTs at 5 μg/ml induced the expression and secretion of TGF-β1 from fibroblasts (Fig. 10c). At both 2 and 5 μg/ml concentrations, MWCNTs induced the active form of TGF-β1 (25 kDa) detected by immunoblotting (Fig. 10d). Thus, MWCNTs stimulate the expression and secretion of both OPN and TGF-β1, and the activation of TGF-β1, in lung fibroblasts directly.

**Fig. 11:**
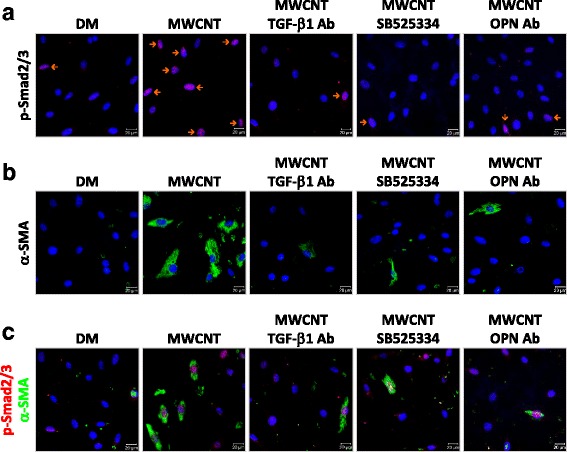
Activation of TGF-β1 signaling and stimulation of myofibroblast differentiation by OPN in cultured lung fibroblasts exposed to MWCNTs. Cells were treated with DM, 5 μg/ml MWCNTs, or 5 μg/ml MWCNTs with TGF-β1 neutralizing antibodies, TGF-β receptor inhibitor SB525334, or OPN neutralizing antibodies for 24 h. (**a**) Expression and nuclear localization of p-Smad2/3 were examined by immunofluorescence (red). Blue indicates nuclear staining (scale bar: 20 μm). Cells positive for nuclear p-Smad2/3 (shown in purple color on the images from the overlapping of red and blue signals) are marked by orange arrows. (**b**) Expression of α-SMA was examined by immunofluorescence (green). Blue indicates nuclear staining (scale bar: 20 μm). (**c**) Expression and nuclear localization of p-Smad2/3 in myofibroblasts (α-SMA positive) and fibroblasts (α-SMA negative) were examined by double immunofluorescence staining of p-Smad2/3 (red) and α-SMA (green). Blue indicates nuclear staining (scale bar: 20 μm)

Activation of TGF-β1 signaling by MWCNTs was examined in relation to the interplay among TGF-β1, type I TGF-β receptor, and OPN in cultured lung fibroblasts. Immunofluorescence staining revealed that MWCNTs remarkably elevated the level of p-Smad2/3 and the number of p-Smad2/3 positive cells, which are indicative of TGF-β1 activation (Fig. 11a). Furthermore, elevation of p-Smad2/3 by MWCNTs was inhibited by TGF-β1 neutralizing antibodies and by a type I TGF-β receptor inhibitor, SB525334, demonstrating that activation of TGF-β signaling by MWCNTs requires TGF-β1 and the type I TGF-β receptor. Importantly, induction of p-Smad2/3 by MWCNTs was also inhibited by OPN neutralizing antibodies. This result indicates that OPN plays a critical role in the activation of TGF-β1 by MWCNTs via the type I TGF-β receptor, thus providing direct evidence to support a cross-interaction between OPN and TGF-β1 signaling in fibroblastic cells upon MWCNT exposure.

Whether MWCNT-induced OPN promotes fibroblast-to-myofibroblast differentiation was examined in vitro. Exposure to MWCNTs markedly increased α-SMA expression and the number of α-SMA positive cells from cultured primary fibroblasts, indicating that MWCNTs stimulate myofibroblast differentiation from fibroblasts directly (Fig. 11b). Treatment with TGF-β1 neutralizing antibodies or SB525334 blocked MWCNT-stimulated elevation of α-SMA expression (Fig. 11b). Moreover, double immunofluorescence demonstrated a high level of p-Smad2/3 in α-SMA+ myofibroblasts under MWCNT exposure and this co-expression of p-Smad2/3 and α-SMA was inhibited by treatment with TGF-β1 neutralizing antibodies or SB525334 (Fig. 11c). These results support a critical role of TGF-β1 signaling in myofibroblast formation induced by MWCNTs. Treatment with OPN neutralizing antibodies also markedly inhibited MWCNT-induced expression of α-SMA (Fig. 11b) as well as MWCNT-induced co-expression of p-Smad2/3 and α-SMA (Fig. 11c). These inhibitory effects by OPN neutralizing antibodies were similar in extent to those by TGF-β1 neutralizing antibodies and TGF-β receptor inhibitor SB525334. Together with the finding that OPN neutralizing antibodies inhibit the activation of TGF-β1 signaling revealed in Fig. 11a, these results suggest that OPN stimulates myofibroblast differentiation directly and this stimulatory effect is mediated through TGF-β1 signaling.

**Fig. 12:**
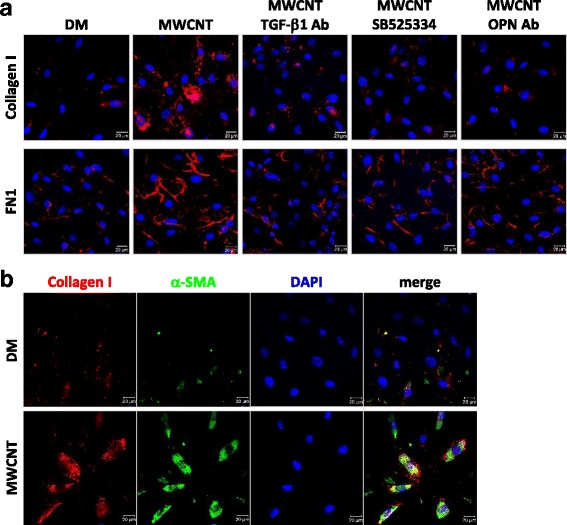
Regulation of fibrotic protein expression by OPN and TGF-β1 in lung fibroblasts exposed to MWCNTs. Cells were treated with DM, 5 μg/ml MWCNTs, or 5 μg/ml MWCNTs with TGF-β1 neutralizing antibodies, TGF-β receptor inhibitor SB525334, or OPN neutralizing antibodies for 24 h. (**a**) Levels of Collagen I (red) and FN1 (red) were examined by immunofluorescence. Blue indicates nuclear staining (scale bar: 20 μm). (**b**) Induction of Collagen I in myofibroblasts by MWCNTs was examined by double immunofluorescence staining of Collagen I (red) and α-SMA (green). Blue indicates nuclear staining (scale bar: 20 μm)

To further corroborate the function of OPN and activation of TGF-β1 in fibrosis induced by MWCNTs, expression of Collagen I and FN1 in cultured primary lung fibroblasts was analyzed. Immunofluorescence staining revealed evident induction of Collagen I and FN1 (Fig. 12a) and the co-expression of Collagen I and α-SMA (Fig. 12b) by MWCNTs. Treatment with OPN neutralizing antibodies markedly reduced the induction of Collagen I and FN1 by MWCNTs similarly to treatment with TGF-β1 neutralizing antibodies or SB525334 (Fig. 12a). These results further support the notion that OPN promotes the fibroblastic response to MWCNTs through the interaction with TGF-β1 signaling.

## Discussion

**Fig. 13:**
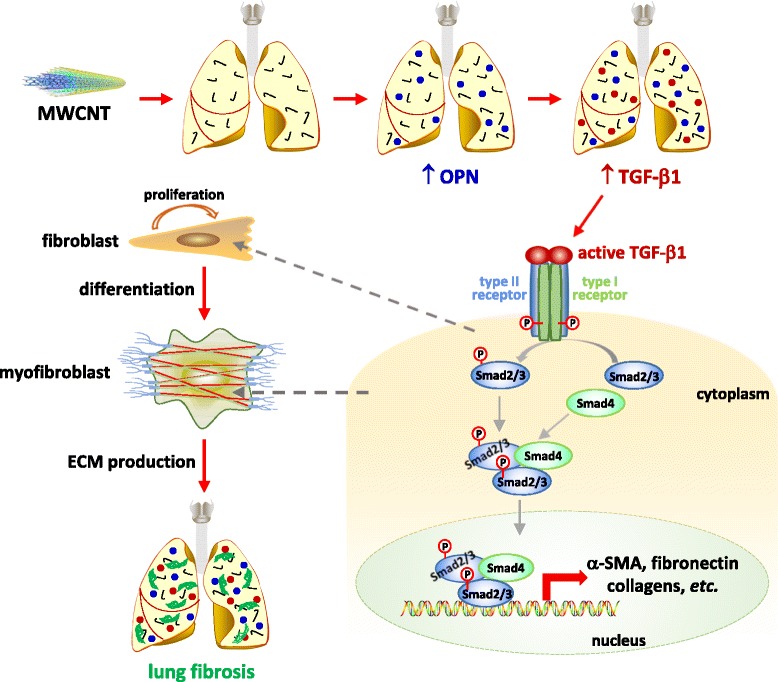
A working model of induced lung fibrosis through OPN. Pulmonary exposure to MWCNTs induces the expression and secretion of OPN in the lungs. Increased OPN stimulates the expression and activation of TGF-β1, and promotes the activation of Smad-dependent TGF-β1 signaling, indicated by phosphorylation and nuclear translocation of Smad2/3, in fibroblasts and myofibroblasts. The activation of Smad-dependent TGF-β1 signaling in fibroblasts induces fibroblast-to-myofibroblast differentiation; and the activation of Smad-dependent TGF-β1 signaling in myofibroblasts results in excessive production and deposition of ECM proteins in lung tissues. Through this signaling cascade, OPN plays an important role in the initiation and progression of MWCNT-induced lung fibrosis

Numerous studies on experimental animals demonstrate that exposure to CNTs potently induces lung fibrosis; moreover, CNT-induced lung fibrosis exhibits a considerable similarity to induced or idiopathic lung fibrosing diseases in humans, prompting exploration of the underlying mechanisms of CNT-induced lung fibrosis [8]. We previously reported that MWCNTs stimulate active gene transcription programs in the lungs that in part account for CNT-induced fibrotic effects [6, 53–55]. Our current study demonstrates that OPN, a multifunctional cytokine and matrix protein, is significantly and persistently induced by MWCNTs in the lungs to a high level; furthermore, induced OPN plays a critical role in MWCNT-induced lung fibrosis through promotion of fibroblast activation and myofibroblast differentiation. At the molecular level, OPN is required for the induction and activation of TGF-β1 signaling in vivo and in cultured primary lung fibroblasts, indicating that OPN exerts its pro-fibrotic effects by regulating fibroblast and myofibroblast functions through the TGF-β1 pathway. These findings allow us to propose a working model, in which the marked and persistent elevation of OPN expression and activation in the lungs by MWCNTs stimulates the TGF-β1 signaling that in turn boosts myofibroblast formation and functionalization leading to fibrosis development (Fig. 13). Our study therefore provides a new molecular insight into the understanding of the initiation and progression of lung fibrosis induced by MWCNTs. The findings also suggest a potential to use OPN as a biomarker for detecting CNT exposure and as a therapeutic target for treating fibrotic lung diseases.

Elevated expression of OPN has been detected in several animal models of induced lung fibrosis, such as those triggered by bleomycin, silica and asbestos, and in human fibrosing diseases, such as IPF and liver cirrhosis [23–34]. Recent reports also show that OPN was induced by chronic exposure to CNTs including both SWCNTs and MWCNTs of various types [35–40]. In view of the anticipated multifold functions of OPN in fibrosis development, a comprehensive profiling of OPN expression in the lungs exposed to CNTs is warranted but was lacking. Therefore, we first examined the regulation of OPN during the acute and chronic phases of the response to MWCNTs, i.e., from day 1 to day 56 post-exposure. The data reveal a marked induction of OPN expression and secretion in both the lung tissues and the BAL fluid by MWCNTs; induction occurred as early as day 1 and persisted up to day 56 post-exposure (Figs. 1 and 2a). MWCNTs potently induced OPN in the BAL at a single low dose of 5 μg on day 7 post-exposure (Fig. 2b). Therefore, OPN is strongly induced in both the acute and chronic responses to MWCNT exposure. This high and persistent induction of OPN suggests a possible role of OPN in both the initiation and progression of MWCNT-induced lung fibrosis.

To investigate the functional impact of OPN induction on MWCNT-induced lung fibrosis, we compared lung fibrotic phenotypes of Opn KO mice with those of WT. Opn KO mice displayed evidently reduced lung fibrosis compared with WT, indicated by attenuated fibrotic focus formation and ECM deposition during both the acute and chronic responses, demonstrating that OPN indeed plays an important role in both the early and late stages of MWCNT-induced fibrosis development (Figs. 3 and 4). Sabo-Attwood et al. [26] reported that Opn mRNA was up-regulated in bronchiolar epithelial cells of mice exposed to asbestos fibers by inhalation. Opn KO in mice led to reduced eosinophilia in the BAL fluid, less inflammation in lung tissues, decreased mucin secretion from the airways, and attenuated induction of fibrotic matrix gene expression, compared with WT upon asbestos exposure. These observations demonstrate the pro-inflammatory and pro-fibrotic activities of OPN in asbestos-exposed lungs, which is in agreement with the findings of the current study on lung fibrosis, albeit different inducers, endpoints, and cell populations were pursued in the two studies. These findings suggest there exist common functions and modes of action of OPN in CNT and asbestos-induced lung lesions to some extent.

Fibroblastic focus formation is often observed in CNT-induced lung fibrosis where fibroblasts and myofibroblasts are major effector cells with multiple roles in matrix production and remodeling [8]. Therefore, we examined fibroblast accumulation and myofibroblast formation. Significantly increased number of fibroblasts and levels of fibroblast marker proteins, such as Hsp47, Vimentin, and FSP1, were detected in MWCNT-exposed WT lungs, but these effects were markedly attenuated in Opn KO lungs (Fig. 5). Thus, MWCNTs induce fibroblast accumulation in lung fibrotic foci in an OPN-dependent manner. Myofibroblasts produce excessive amounts of ECM proteins and exert a high contractile activity during fibrotic matrix remodeling and scar formation [9, 10, 56]. We posited that OPN stimulates myofibroblast transformation and activation in CNT-induced lung fibrosis. By detecting two markers of myofibroblasts, α-SMA and PDGFR-β, we showed that MWCNTs potently induced the formation of myofibroblasts in WT lungs, especially in fibrotic foci; but the induction was significantly repressed in Opn KO lungs, indicating that OPN is required for myofibroblast differentiation in MWCNT-exposed lungs (Fig. 6). Reduction in MWCNT-induced production and deposition of ECM proteins was observed in Opn KO lungs, which further confirms the promoting function of OPN in myofibroblast transformation and functionalization (Fig. 4). Therefore, OPN promotes MWCNT-induced lung fibrosis in part through boosting myofibroblast formation and function.

TGF-β1 plays multiple roles in the pathogenesis of lung fibrosis, including promotion of fibroblast proliferation, fibroblast-to-myofibroblast differentiation, and ECM synthesis. As such, it is regarded as one of the most important fibrogenic mediators [16, 17]. Increased expression of TGF-β1 was observed in several fibrotic lung models and diseases, such as bleomycin-induced lung fibrosis and IPF. Notably, TGF-β1 was induced by CNTs in the lungs and in cultured cells in a number of studies, suggesting a role of TGF-β1 in CNT-induced fibrogenic response [8]. Moreover, recent studies have linked OPN to the regulation of TGF-β1. For example, loss of OPN reduced TGF-β1-induced myofibroblast differentiation in cultured cardiac or dermal fibroblasts; OPN enhanced wound healing in cultured liver progenitor cells by modulating TGF-β1 signaling; and OPN neutralization abrogated liver fibrogenesis in mice [57, 58]. We therefore determined whether OPN modulates TGF-β1 signaling in CNT-induced lung fibrosis. We found that MWCNTs remarkably stimulated the expression and activation of TGF-β1 and TGF-β1 signaling in the lungs during both the acute and chronic phase responses in an OPN-dependent manner (Figs. 7 and 8). These data provide the in vivo evidence to support a function of OPN in promoting TGF-β1 signaling during MWCNT-induced lung fibrosis.

Upon stimulation, active TGF-β1 binds to its receptors to form a complex consisting of one TGF-β1 homodimer, two type I receptors, and two type II receptors on the cell surface, which leads to the activation of the Smad-dependent pathway to induce the transcription of fibrotic genes, such as the genes encoding α-SMA, collagens and fibronectin (Fig. 13). Activation of TGF-β1 signaling promotes the fibroblast-to-myofibroblast differentiation and excessive production and secretion of fibrotic ECM proteins from myofibroblasts. In this study, we found that MWCNTs dramatically induced the phosphorylation of Smad2/3 and the translocation of p-Smad2/3 to the nucleus in fibroblasts and myofibroblasts in WT lungs, which were markedly attenuated in both cell types in Opn KO lungs (Fig. 9). This study demonstrates and visualizes the activation of TGF-β1 signaling in fibroblasts and myofibroblasts in MWCNT-exposed lungs. Moreover, it reveals that OPN enhances the activation of TGF-β1 signaling in fibroblasts and myofibroblasts in the lungs in response to MWCNT exposure, thereby providing novel evidence supporting the interplay between OPN and TGF-β1 in lung fibrosis.

The stimulating functions of OPN on TGF-β1 induction and signaling in lung fibroblasts were further confirmed by several lines of evidence obtained in vitro using primary fibroblasts derived from mouse lungs. First, MWCNTs were shown to induce the expression and activation of OPN and TGF-β1, as well as the activation of TGF-β1 signaling, in cultured lung fibroblasts (Figs. 10 and 11a). Second, MWCNTs stimulated the differentiation of myofibroblasts from fibroblasts, indicated by induced expression of α-SMA and increased production of Collagen I and FN1 in cells where TGF-β1 signaling was activated; moreover, induction of α-SMA, Collagen I, and FN1 was blocked by co-treating with TGF-β1 neutralizing antibodies or type I TGF-β receptor inhibitor SB525334, confirming the critical role of TGF-β1 in MWCNT-induced myofibroblast formation and function (Figs. 11b, c and 12a). Third, co-treating the cells with MWCNTs and OPN neutralizing antibodies markedly attenuated the activation of TGF-β1 signaling in parallel with significant inhibition of α-SMA expression and Collagen I and FN1 production, demonstrating that OPN is required for optimal activation of TGF-β1 signaling, differentiation of myofibroblasts, and deposition of fibrous ECM induced by MWCNTs (Figs. 11 and 12a). Together, these in vitro results complement the in vivo findings by demonstrating that OPN directly regulates myofibroblast formation and function via TGF-β1 signaling in fibroblastic cells.

OPN is widely expressed in tissues under physiological conditions and its expression and secretion are highly induced in a variety of tissue injury and disease states besides induced lung fibrosis discussed in the current study. Our finding that OPN expression is drastically induced by MWCNTs in both the acute and chronic responses to exposure is in agreement with the rapid-onset and persistence of MWCNT-induced lung fibrosis. This feature of OPN induction therefore suggests the potential for OPN to serve as a biomarker for monitoring fibrogenic nanoparticle exposure and as a therapeutic target for treating induced lung fibrosis.

## Conclusion

The findings from the current study reveal an OPN-promoted, TGF-β1-driven, and myofibroblast-mediated mechanism for MWCNT-induced lung fibrosis. The study demonstrates that OPN is highly induced by MWCNTs to activate Smad-dependent TGF-β1 signaling and to stimulate myofibroblast transformation and functionalization, through which OPN functions as a pro-fibrotic factor to boost MWCNT-induced lung fibrosis. Therefore, our study highlights an OPN-dependent cellular and molecular mechanism for MWCNT-triggered fibrotic response in mouse lungs. Moreover, our data support a critical interplay between OPN and TGF-β1 signaling in the development of CNT-induced lung fibrosis.

## Abbreviations

AKT: v-akt murine thymoma viral oncogene homolog
BAL: Bronchoalveolar lavage
CNT: Carbon nanotube
DM: Dispersion medium
ECM: Extracellular matrix
EMT: Epithelial to mesenchymal transition
FN1: Fibronectin
FSP1: Fibroblast specific protein 1
Gapdh: Glyceraldehyde 3-phosphate dehydrogenase
Hsp47: Heat shock protein 47
IPF: Idiopathic pulmonary fibrosis
KO: Knockout
MWCNT: Multi-walled carbon nanotube
OPN: Osteopontin
PDGFR-β: Platelet-derived growth factor receptor-β
PI3K: Phosphoinositide 3-kinase
Smad: Sma and Mad related family
SPP1: Secreted phosphoprotein 1
SWCNT: Single-walled carbon nanotube
TGF-β1: Transforming growth factor-β1
WT: Wild-type
α-SMA: α-smooth muscle actin
